#  Infantile Hypertrophic Pyloric Stenosis in Postoperative Esophageal Atresia with Tracheoesophageal Fistula

**Published:** 2015-07-01

**Authors:** Hassan R A A, Choo Y U, Noraida R, Rosida I

**Affiliations:** 1 Department of Pediatric Surgery, School of Medical Sciences, Universiti Sains Malaysia; 2 Department of Surgery, Universiti Sains Malaysia; 3 Department of Neonatology, Universiti Sains Malaysia; 4 Department of Pediatric, Universiti Sains Malaysia

**Keywords:** Esophageal atresia, Tracheoesophageal fistula, Infantile hypertrophic pyloric stenosis

## Abstract

Development of infantile hypertrophic pyloric stenosis during postoperative period in EA with TEF is rare. Postoperative vomiting or feeding intolerance in EA is more common which is due to esophageal stricture, gastroesophageal reflux and esophageal dysmotility. A typical case of IHPS also presents with non-bilious projectile vomiting at around 3-4 weeks of life. The diagnosis of infantile hypertrophic pyloric stenosis in this subset is usually delayed because of its rarity. We report a case of IHPS in postoperative EA and emphasize on high index of suspicion to avoid any delay in diagnosis with its metabolic consequences.

## CASE REPORT

A 2450gm baby boy was born to a primigravida mother at 39 weeks of gestational age at our institution through spontaneous vaginal delivery. The pregnancy was uneventful except detection of polyhydramnios at 28th week of gestation. Apgar score at 1st minute was 8. He had excessive salivation. The diagnosis of esophageal atresia (EA) was confirmed, as the orogastric tube could not be passed into the stomach. He was also noted to have malformed left thumb and anorectal malformation. Abdominal ultrasonography revealed absent left kidney. Echocardiogram showed a moderate sized patent ductus arteriosus (PDA) with left aortic arch. On his 2nd day of life, ligation-division of fistula and primary esophageal anastomosis performed via right thoracotomy; a sigmoid colostomy was also done. The baby remained ventilated and noticed to have saliva in chest drain on 5th postoperative day. The anastomotic leak healed on conservative management. The baby had stormy course during his NICU stay. He was treated for sepsis and fungal septicemia. The baby was discharged from hospital at D-31 when he was taking full oral feeds. In his follow up visit in outpatient clinic at 6 weeks after surgery, he was reported to have feed intolerance. A contrast esophagogram at 7th week revealed anastomotic stricture and reflux of contrast above the anastomosis. He was booked for esophagoscopy. In the meantime, a week later at the age of 8 weeks, he presented with large amount of milk-curd vomiting. An abdominal x-ray showed grossly dilated stomach. Diagnosis of infantile hypertrophic pyloric stenosis (IHPS) was suspected and was later confirmed on abdominal ultrasonography; pyloric muscle thickness measured 6.5 mm, pyloric diameter 13.6 mm and canal length 17.0 mm (Fig. 1). An esophagoscopy done under general anesthesia and endoscopic dilatation confirmed an easy and pliable anastomosis. Ramstedt’s Pyloromyotomy was done in the same sitting. The feeds were started next morning. He was discharged home on 3rd postoperative day with the advice for regular follow-up. Unfortunately, the boy was readmitted at 5 months of life with diagnosis of aspiration and fulminant sepsis and he died within short period of time.

**Figure:**
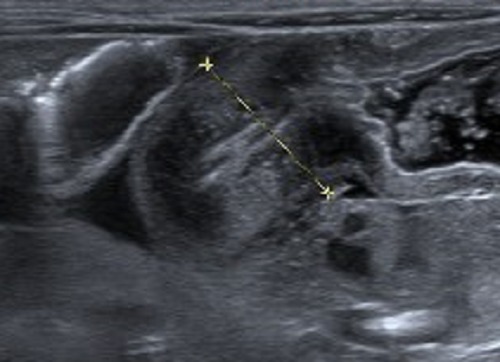
Figure 1: USG Confirmed IHPS

## DISCUSSION

Only 36 cases have been reported in the past literature [1]. The association of IHPS with EA was first reported in 1969 [2]. The absence of classical symptoms and signs of IHPS may be absent or masked after an EA or abdomino-thoracic surgery and this leads to delay in diagnosis. Diagnosis is usually confirmed after radio-imaging; upper Gastro-intestinal contrast study, ultrasonography or both [3,4]. 

In our case, the baby boy had all components of VACTERL association except vertebral anomaly. He had minor anastomotic leakage which was treated conservatively. He developed anastomotic stricture which was initially attributed to his feeding problem. Moreover he had GER. Diagnosis of IHPS was done at 8 weeks of life. The strong suspicion of IHPS came after taking careful history of frequent episodes of milk curd vomiting noticed at 7th week and a dilated stomach on a plain x-ray abdomen. IHPS is so common that its occasional association with EA is probably coincidental. Symptoms of IHPS are easily attributable to the postoperative complication of primary surgery [5].

In contrast to the common complications of EA surgery, IHPS does not come to consideration in postoperative vomiting. It is essential to be aware of increased incidence of IHPS in this group of patients. A high index of suspicion and diagnostic radio-imaging may make an earlier diagnosis and intervention.

## Footnotes

**Source of Support:** Nil

**Conflict of Interest:**

